# Tetra­kis(μ_2_-5-methyl­pyrazine-2-carboxyl­ato)-1:2κ^3^ *N* ^1^,*O*:*O*;2:3κ^3^ *O*:*N* ^1^,*O*;1:2κ^2^ *O*:*O*′;3:4κ^2^ *O*:*O*′-octa­octyl-1κ^2^ *C*,2κ^2^ *C*,3κ^2^ *C*,4κ^2^ *C*-di-μ_3_-oxido-1:2:3κ^3^ *O*;1:3:4κ^3^ *O*-tetra­tin(IV)

**DOI:** 10.1107/S1600536809047345

**Published:** 2009-11-14

**Authors:** Zhongjun Gao, Fahui Li

**Affiliations:** a Department of Chemistry, Jining University, Shandong 273155, People’s Republic of China; b Marine Drug and Food Institute, Ocean University of China, Qingdao 266003, People’s Republic of China

## Abstract

The title compound, [Sn_4_(C_8_H_17_)_8_O_2_(C_6_H_5_N_2_O_2_)_4_], is a tetra­nuclear Sn^IV^ complex, built up by inversion symmetry around the central Sn_2_O_2_ ring. The Sn^IV^ coordination geometries are distorted SnO_3_C_2_ trigonal-bipyramidal and distorted SnO_4_C_2_ octa­hedral. The three-coordinate μ_3_-oxido bridging O atom in the Sn_2_O_2_ ring is attached to three Sn atoms. All non-H atoms, with the exception of the Sn-bonded octyl groups, lie approximately on a non-crystallographic mirror plane.

## Related literature

For biological activity of organotin derivatives of carboxylic acid ligands, see: Gielen *et al.* (1988 ▶). For related μ_3_-oxo bridged Sn^IV^ structures, see: Vollano *et al.* (1984 ▶); Yin *et al.* (2003 ▶).
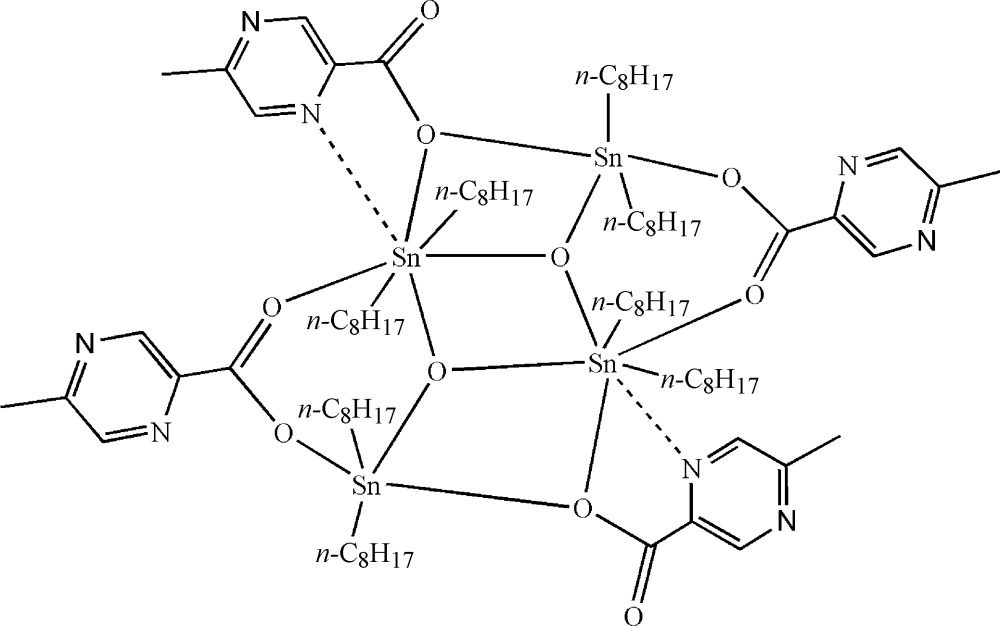


## Experimental

### 

#### Crystal data

[Sn_4_(C_8_H_17_)_8_O_2_(C_6_H_5_N_2_O_2_)_4_]
*M* *_r_* = 1960.97
Triclinic, 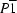

*a* = 12.406 (4) Å
*b* = 13.282 (4) Å
*c* = 16.223 (5) Å
α = 76.623 (4)°
β = 73.361 (4)°
γ = 86.522 (3)°
*V* = 2491.7 (13) Å^3^
*Z* = 1
Mo *K*α radiation
μ = 1.05 mm^−1^
*T* = 295 K
0.63 × 0.54 × 0.49 mm

#### Data collection

Bruker SMART CCD diffractometer
Absorption correction: multi-scan (*SADABS*; Sheldrick, 1996 ▶) *T*_min_ = 0.602, *T*_max_ = 0.623
12764 measured reflections
8613 independent reflections
4935 reflections with *I* > 2σ(*I*)
*R*_int_ = 0.041

#### Refinement

*R*[*F* ^2^ > 2σ(*F* ^2^)] = 0.064
*wR*(*F* ^2^) = 0.223
*S* = 1.11
8613 reflections
496 parameters
H-atom parameters constrained
Δρ_max_ = 3.10 e Å^−3^
Δρ_min_ = −1.14 e Å^−3^

### 

Data collection: *SMART* (Bruker 1998 ▶); cell refinement: *SAINT* (Bruker 1998 ▶); data reduction: *SAINT*; program(s) used to solve structure: *SHELXS97* (Sheldrick, 2008 ▶); program(s) used to refine structure: *SHELXL97* (Sheldrick, 2008 ▶); molecular graphics: *SHELXTL* (Sheldrick, 2008 ▶); software used to prepare material for publication: *SHELXL97* and *PLATON* (Spek, 2009 ▶).

## Supplementary Material

Crystal structure: contains datablocks I, global. DOI: 10.1107/S1600536809047345/si2220sup1.cif

Structure factors: contains datablocks I. DOI: 10.1107/S1600536809047345/si2220Isup2.hkl

Additional supplementary materials:  crystallographic information; 3D view; checkCIF report

## Figures and Tables

**Table 1:** Selected bond lengths (Å)

Sn1—O5	1.993 (6)
Sn1—C21	2.133 (16)
Sn1—C13	2.135 (13)
Sn1—O4^i^	2.158 (7)
Sn1—O1	2.202 (6)
Sn2—C37	2.090 (12)
Sn2—C29	2.093 (13)
Sn2—O5^i^	2.111 (6)
Sn2—O5	2.151 (6)
Sn2—O1	2.455 (7)
Sn2—O3	2.472 (7)

Symmetry code: (i) 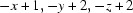.

**Fig. 1.**
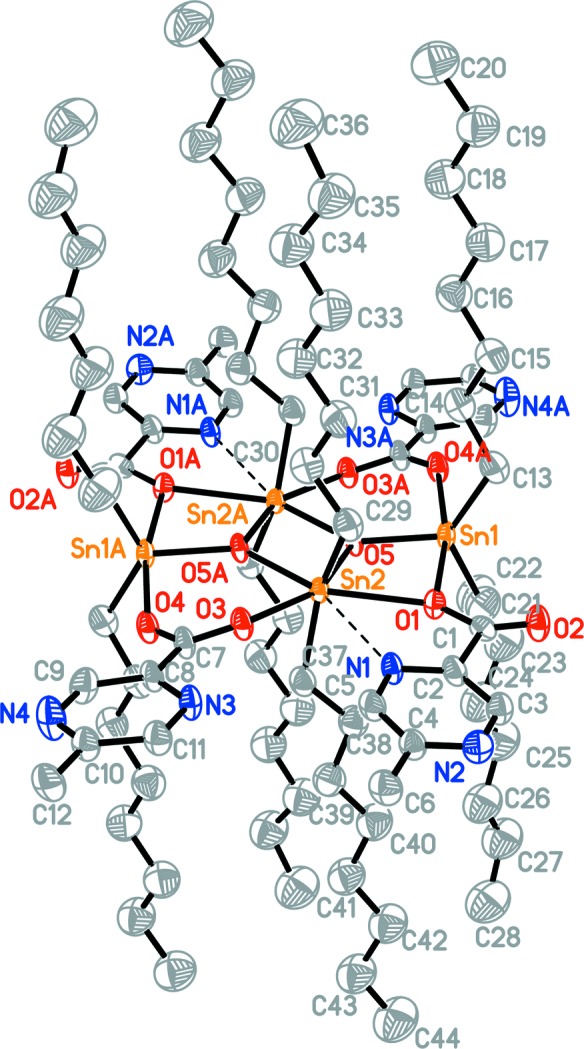
The molecular structure of (I) with 30% displacement ellipsoids (H atoms omitted for clarity) and the short Sn···N contacts indicated by dashed lines. Symmetry codes as in Table 1.

**Fig. 2.**
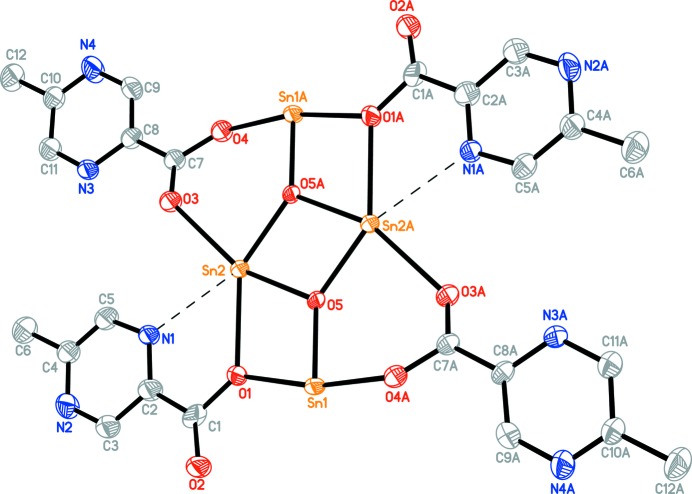
The same view as in Figure 1, but the n-capryl groups have been omitted for clarity.

## supplementary crystallographic information

### Comment

Self-assembled organotin derivatives of carboxylic acid ligands have been extensively studied due to their biological activities (Gielen, *et al.*, 1988). 2-Methylpyrazine-5-carboxylic acid is a good bridging ligand that can sometimes be used to generate unexpected and interesting coordination polymers, and small changes in experimental conditions can lead to very different architectures.

The title compound, (Fig. 1), is a tetranuclear tin(IV) complex containing a total of 110 non-H atoms. The molecule is centrosymmetric with a central core Sn_2_O_2_; the structure is similar to those seen previously in resemble compounds (Yin *et al.*, 2003). The µ_3_-bridging O5 atom in the Sn_2_O_2_ ring is also attached to a capryl_2_SnO_2_ unit (capryl is the trivial name of the octyl group). In addition, the carboxylate group coordinates to two Sn atoms in a bridging mode. Sn—O and Sn—C bond lenghts are shown in Table 1.

The geometries of both the exocyclic Sn atoms are distorted trigonal- bipyramidal. For the Sn1, atoms O1 and O7 are in axial positions [O1—Sn1—O4 = 168.0 (3)°] and the C atoms of the two capryl groups and O5 are in equatorial positions. The sum of the equatorial C—Sn—C and O—Sn—C angles is 339°, indicating a significant distortion from coplanarity for these atoms.

The geometry around the endocyclic atom Sn2 is different from that of Sn1 and is a distorted octahedron. Here, O3 and O5 are in axial positions [O3—Sn2—O5 =158.9 (3)°] and the C atoms of the two capryl groups, O5^i^ [symmetry code: -*x* + 1, -*y* + 2, -*z* + 2] and O1 are in equatorial positions. The sum of the equatorial C—Sn—C and O—Sn—C angles is 353.7°, indicating a significant distortion from coplanarity for these atoms. This distortion may arise because of a short Sn2···N1 contact of 2.829 (6)Å (sum of the van der Waals radii = 3.81 Å). Related µ_3_ oxo-bridhed tin(IV) ladder structures were reported by Vollano *et al.*, 1984) (Fig. 2).

### Experimental

A mixture of dicapryltin oxide (2.0 mmol,0.722 g) and 2-methylpyrazine-5-carboxylic acid (2.0 mmol, 0.276 g) in methanol (80 ml) was heated under reflux for 8 h. The obtained clear solution was evaporated under vacuum. The product was crystallized from a mixture of dichloromethane/ethanol (1:1) to yield blocks of (I). Yield 0.804 g, 82%, m.p. 368 K, analysis, calculated for C_88_H_156_N_8_O_10_Sn_4_: C 53.90, H, 8.02; N 5.71%; found: C 53.92, H 8.09, N, 5.76%.

### Refinement

H atoms were positioned geometrically [0.93 (CH), 0.97 (CH_2_) and 0.96 (CH_3_) Å] and constrained to ride on their parent atoms with *U*_iso_(H) = 1.2(1.5 for methyl)*U*_eq_(C/N).

### Crystal data

**Table:**

[Sn_4_(C_8_H_17_)_8_O_2_(C_6_H_5_N_2_O_2_)_4_]	*Z* = 1
*M**_r_* = 1960.97	*F*(000) = 1020
Triclinic, *P*1	*D*_x_ = 1.307 Mg m^−^^3^
Hall symbol: -P 1	Mo *K*α radiation, λ = 0.71073 Å
*a* = 12.406 (4) Å	Cell parameters from 3961 reflections
*b* = 13.282 (4) Å	θ = 2.2–26.6°
*c* = 16.223 (5) Å	µ = 1.05 mm^−^^1^
α = 76.623 (4)°	*T* = 295 K
β = 73.361 (4)°	Block, colourless
γ = 86.522 (3)°	0.63 × 0.54 × 0.49 mm
*V* = 2491.7 (13) Å^3^	

### Data collection

**Table:**

Bruker SMART CCD diffractometer	8613 independent reflections
Radiation source: fine-focus sealed tube	4935 reflections with *I* > 2σ(*I*)
graphite	*R*_int_ = 0.041
φ and ω scans	θ_max_ = 25.0°, θ_min_ = 1.6°
Absorption correction: multi-scan (*SADABS*; Sheldrick, 1996)	*h* = −11→14
*T*_min_ = 0.602, *T*_max_ = 0.623	*k* = −15→15
12764 measured reflections	*l* = −19→18

### Refinement

**Table:**

Refinement on *F*^2^	Primary atom site location: structure-invariant direct methods
Least-squares matrix: full	Secondary atom site location: difference Fourier map
*R*[*F*^2^ > 2σ(*F*^2^)] = 0.064	Hydrogen site location: inferred from neighbouring sites
*wR*(*F*^2^) = 0.223	H-atom parameters constrained
*S* = 1.11	*w* = 1/[σ^2^(*F*_o_^2^) + (0.0784*P*)^2^ + 14.9991*P*] where *P* = (*F*_o_^2^ + 2*F*_c_^2^)/3
8613 reflections	(Δ/σ)_max_ = 0.001
496 parameters	Δρ_max_ = 3.10 e Å^−^^3^
0 restraints	Δρ_min_ = −1.14 e Å^−^^3^

### Special details

**Table:**

Geometry. All e.s.d.'s (except the e.s.d. in the dihedral angle between two l.s. planes) are estimated using the full covariance matrix. The cell e.s.d.'s are taken into account individually in the estimation of e.s.d.'s in distances, angles and torsion angles; correlations between e.s.d.'s in cell parameters are only used when they are defined by crystal symmetry. An approximate (isotropic) treatment of cell e.s.d.'s is used for estimating e.s.d.'s involving l.s. planes.
Refinement. Refinement of *F*^2^ against ALL reflections. The weighted *R*-factor *wR* and goodness of fit *S* are based on *F*^2^, conventional *R*-factors *R* are based on *F*, with *F* set to zero for negative *F*^2^. The threshold expression of *F*^2^ > σ(*F*^2^) is used only for calculating *R*-factors(gt) *etc*. and is not relevant to the choice of reflections for refinement. *R*-factors based on *F*^2^ are statistically about twice as large as those based on *F*, and *R*- factors based on ALL data will be even larger.

### Fractional atomic coordinates and isotropic or equivalent isotropic displacement parameters (Å^2^)

**Table:**

	*x*	*y*	*z*	*U*_iso_*/*U*_eq_	
Sn1	0.75921 (6)	1.02020 (5)	1.00407 (5)	0.0425 (3)	
Sn2	0.54331 (5)	0.88620 (5)	0.97148 (5)	0.0352 (2)	
N1	0.6754 (7)	0.7190 (6)	0.9181 (6)	0.048 (2)	
N2	0.8219 (9)	0.5630 (7)	0.8661 (7)	0.064 (3)	
N3	0.3417 (7)	0.5913 (7)	0.9200 (7)	0.053 (3)	
N4	0.1270 (10)	0.5904 (8)	0.9034 (9)	0.082 (4)	
O1	0.7472 (6)	0.8839 (5)	0.9515 (5)	0.0458 (18)	
O2	0.9195 (7)	0.8183 (6)	0.9440 (6)	0.065 (2)	
O3	0.4103 (6)	0.7570 (5)	0.9680 (5)	0.051 (2)	
O4	0.2668 (6)	0.8471 (5)	0.9371 (5)	0.052 (2)	
O5	0.6013 (5)	1.0223 (5)	0.9969 (5)	0.0377 (16)	
C1	0.8261 (10)	0.8085 (8)	0.9410 (8)	0.050 (3)	
C2	0.7819 (10)	0.7240 (9)	0.9119 (8)	0.055 (3)	
C3	0.8574 (11)	0.6445 (9)	0.8875 (9)	0.060 (3)	
H3	0.9324	0.6488	0.8863	0.072*	
C4	0.7144 (10)	0.5611 (9)	0.8713 (8)	0.056 (3)	
C5	0.6447 (10)	0.6354 (9)	0.8977 (8)	0.057 (3)	
H5	0.5690	0.6284	0.9021	0.068*	
C6	0.6730 (12)	0.4696 (10)	0.8488 (10)	0.073 (4)	
H6A	0.7351	0.4253	0.8308	0.109*	
H6B	0.6389	0.4933	0.8017	0.109*	
H6C	0.6185	0.4318	0.8998	0.109*	
C7	0.3250 (9)	0.7600 (8)	0.9431 (8)	0.051 (3)	
C8	0.2775 (9)	0.6749 (8)	0.9274 (8)	0.051 (3)	
C9	0.1718 (11)	0.6705 (9)	0.9178 (9)	0.066 (4)	
H9	0.1274	0.7291	0.9218	0.079*	
C10	0.1861 (11)	0.5135 (9)	0.8960 (9)	0.059 (3)	
C11	0.2959 (10)	0.5123 (9)	0.9025 (9)	0.061 (3)	
H11	0.3396	0.4540	0.8943	0.073*	
C12	0.1371 (12)	0.4235 (10)	0.8802 (10)	0.075 (4)	
H12A	0.1070	0.3754	0.9354	0.112*	
H12B	0.1944	0.3902	0.8420	0.112*	
H12C	0.0780	0.4464	0.8530	0.112*	
C13	0.7885 (12)	0.9313 (11)	1.1232 (9)	0.073 (4)	
H13A	0.7954	0.8591	1.1200	0.087*	
H13B	0.8596	0.9529	1.1275	0.087*	
C14	0.6980 (13)	0.9405 (12)	1.2060 (10)	0.081 (4)	
H14A	0.6257	0.9258	1.1993	0.097*	
H14B	0.6965	1.0114	1.2126	0.097*	
C15	0.7133 (13)	0.8685 (12)	1.2899 (10)	0.086 (5)	
H15A	0.7168	0.7976	1.2831	0.104*	
H15B	0.7843	0.8846	1.2981	0.104*	
C16	0.6188 (15)	0.8776 (13)	1.3717 (11)	0.096 (5)	
H16A	0.6163	0.9483	1.3790	0.116*	
H16B	0.5477	0.8629	1.3630	0.116*	
C17	0.6319 (16)	0.8052 (14)	1.4551 (12)	0.103 (5)	
H17A	0.7018	0.8213	1.4650	0.124*	
H17B	0.6366	0.7347	1.4474	0.124*	
C18	0.5347 (16)	0.8129 (14)	1.5359 (12)	0.110 (6)	
H18A	0.4646	0.7974	1.5262	0.132*	
H18B	0.5305	0.8830	1.5446	0.132*	
C19	0.5503 (17)	0.7374 (16)	1.6189 (13)	0.121 (7)	
H19A	0.5559	0.6673	1.6100	0.145*	
H19B	0.6194	0.7538	1.6297	0.145*	
C20	0.4504 (19)	0.7450 (17)	1.6984 (14)	0.145 (8)	
H20A	0.4610	0.6981	1.7501	0.218*	
H20B	0.3824	0.7273	1.6879	0.218*	
H20C	0.4452	0.8145	1.7070	0.218*	
C21	0.8774 (14)	1.0941 (13)	0.8843 (11)	0.098 (5)	
H21A	0.9513	1.0669	0.8859	0.117*	
H21B	0.8596	1.0736	0.8360	0.117*	
C22	0.8853 (17)	1.2086 (15)	0.8628 (13)	0.125 (7)	
H22A	0.9007	1.2301	0.9116	0.150*	
H22B	0.8127	1.2366	0.8582	0.150*	
C23	0.9729 (17)	1.2552 (16)	0.7797 (14)	0.127 (7)	
H23A	0.9758	1.3290	0.7758	0.152*	
H23B	1.0453	1.2270	0.7848	0.152*	
C24	0.9587 (17)	1.2402 (16)	0.6965 (14)	0.125 (7)	
H24A	0.8811	1.2543	0.6962	0.150*	
H24B	0.9735	1.1683	0.6935	0.150*	
C25	1.0353 (18)	1.3088 (16)	0.6151 (15)	0.131 (7)	
H25A	1.0141	1.3804	0.6147	0.158*	
H25B	1.1120	1.3010	0.6194	0.158*	
C26	1.0324 (18)	1.2861 (17)	0.5299 (15)	0.131 (7)	
H26A	0.9557	1.2926	0.5260	0.157*	
H26B	1.0554	1.2150	0.5296	0.157*	
C27	1.108 (2)	1.3568 (18)	0.4499 (16)	0.144 (8)	
H27A	1.0823	1.4274	0.4494	0.173*	
H27B	1.1834	1.3527	0.4559	0.173*	
C28	1.111 (2)	1.3339 (19)	0.3636 (17)	0.166 (10)	
H28A	1.1636	1.3801	0.3170	0.249*	
H28B	1.0379	1.3431	0.3547	0.249*	
H28C	1.1350	1.2637	0.3635	0.249*	
C29	0.5414 (11)	0.7814 (10)	1.0901 (9)	0.068 (4)	
H29A	0.5512	0.7128	1.0778	0.082*	
H29B	0.6074	0.7957	1.1062	0.082*	
C30	0.4425 (13)	0.7758 (12)	1.1704 (10)	0.083 (4)	
H30A	0.3759	0.7564	1.1576	0.099*	
H30B	0.4297	0.8438	1.1837	0.099*	
C31	0.4592 (14)	0.6980 (12)	1.2516 (11)	0.089 (5)	
H31A	0.4744	0.6306	1.2374	0.107*	
H31B	0.5249	0.7187	1.2650	0.107*	
C32	0.3609 (15)	0.6884 (13)	1.3323 (11)	0.101 (5)	
H32A	0.2954	0.6660	1.3198	0.122*	
H32B	0.3446	0.7558	1.3463	0.122*	
C33	0.3816 (16)	0.6122 (14)	1.4119 (12)	0.109 (6)	
H33A	0.3944	0.5443	1.3984	0.131*	
H33B	0.4497	0.6327	1.4219	0.131*	
C34	0.2882 (17)	0.6041 (16)	1.4950 (13)	0.121 (6)	
H34A	0.2200	0.5815	1.4865	0.145*	
H34B	0.2742	0.6717	1.5091	0.145*	
C35	0.3176 (18)	0.5265 (16)	1.5727 (14)	0.131 (7)	
H35A	0.3170	0.4565	1.5643	0.157*	
H35B	0.3924	0.5412	1.5745	0.157*	
C36	0.232 (2)	0.5354 (19)	1.6591 (16)	0.169 (10)	
H36A	0.2496	0.4865	1.7071	0.253*	
H36B	0.1580	0.5211	1.6569	0.253*	
H36C	0.2342	0.6042	1.6679	0.253*	
C37	0.5683 (10)	0.9247 (10)	0.8349 (8)	0.061 (3)	
H37A	0.5380	0.8677	0.8202	0.073*	
H37B	0.5208	0.9840	0.8230	0.073*	
C38	0.6816 (11)	0.9497 (11)	0.7698 (9)	0.074 (4)	
H38A	0.7279	0.8882	0.7735	0.088*	
H38B	0.7174	1.0023	0.7858	0.088*	
C39	0.6774 (13)	0.9875 (12)	0.6759 (10)	0.086 (4)	
H39A	0.6394	0.9350	0.6617	0.104*	
H39B	0.6305	1.0487	0.6735	0.104*	
C40	0.7846 (15)	1.0136 (14)	0.6054 (11)	0.104 (5)	
H40A	0.8313	0.9523	0.6058	0.124*	
H40B	0.8238	1.0657	0.6192	0.124*	
C41	0.7724 (15)	1.0529 (15)	0.5138 (12)	0.109 (6)	
H41A	0.7236	1.0052	0.5040	0.131*	
H41B	0.7341	1.1189	0.5121	0.131*	
C42	0.8757 (16)	1.0673 (16)	0.4391 (13)	0.120 (7)	
H42A	0.9127	1.0009	0.4380	0.144*	
H42B	0.9264	1.1131	0.4490	0.144*	
C43	0.8560 (17)	1.1118 (17)	0.3492 (13)	0.127 (7)	
H43A	0.8058	1.0654	0.3394	0.153*	
H43B	0.8178	1.1775	0.3510	0.153*	
C44	0.9569 (19)	1.1282 (19)	0.2737 (15)	0.165 (10)	
H44A	0.9357	1.1556	0.2204	0.247*	
H44B	0.9949	1.0636	0.2701	0.247*	
H44C	1.0062	1.1763	0.2811	0.247*	

### Atomic displacement parameters (Å^2^)

**Table:**

	*U*^11^	*U*^22^	*U*^33^	*U*^12^	*U*^13^	*U*^23^
Sn1	0.0267 (4)	0.0374 (4)	0.0702 (6)	0.0005 (3)	−0.0202 (4)	−0.0176 (4)
Sn2	0.0282 (4)	0.0306 (4)	0.0502 (5)	−0.0008 (3)	−0.0144 (3)	−0.0113 (3)
N1	0.038 (5)	0.040 (5)	0.078 (7)	0.002 (4)	−0.030 (5)	−0.020 (5)
N2	0.065 (7)	0.048 (6)	0.094 (9)	0.010 (5)	−0.029 (6)	−0.038 (6)
N3	0.038 (5)	0.044 (5)	0.085 (8)	0.000 (4)	−0.028 (5)	−0.017 (5)
N4	0.070 (8)	0.049 (6)	0.152 (12)	0.008 (6)	−0.056 (8)	−0.043 (7)
O1	0.037 (4)	0.036 (4)	0.073 (5)	0.004 (3)	−0.021 (4)	−0.020 (4)
O2	0.046 (5)	0.054 (5)	0.113 (8)	0.004 (4)	−0.037 (5)	−0.035 (5)
O3	0.041 (4)	0.044 (4)	0.083 (6)	−0.003 (3)	−0.031 (4)	−0.023 (4)
O4	0.042 (4)	0.044 (4)	0.083 (6)	−0.002 (4)	−0.031 (4)	−0.023 (4)
O5	0.030 (4)	0.027 (3)	0.064 (5)	−0.003 (3)	−0.025 (3)	−0.010 (3)
C1	0.053 (7)	0.050 (7)	0.062 (8)	−0.013 (6)	−0.022 (6)	−0.033 (6)
C2	0.049 (7)	0.057 (7)	0.072 (9)	0.000 (6)	−0.031 (6)	−0.020 (6)
C3	0.051 (8)	0.057 (8)	0.078 (9)	0.001 (6)	−0.024 (7)	−0.019 (7)
C4	0.053 (8)	0.052 (7)	0.072 (9)	−0.001 (6)	−0.025 (7)	−0.021 (6)
C5	0.053 (8)	0.053 (7)	0.077 (9)	−0.007 (6)	−0.027 (7)	−0.024 (7)
C6	0.075 (10)	0.061 (8)	0.093 (11)	−0.008 (7)	−0.032 (8)	−0.027 (8)
C7	0.040 (6)	0.044 (6)	0.082 (9)	−0.002 (5)	−0.031 (6)	−0.023 (6)
C8	0.044 (7)	0.044 (6)	0.079 (9)	−0.007 (5)	−0.029 (6)	−0.026 (6)
C9	0.055 (8)	0.047 (7)	0.099 (11)	0.010 (6)	−0.022 (7)	−0.025 (7)
C10	0.056 (8)	0.046 (7)	0.087 (10)	−0.004 (6)	−0.028 (7)	−0.025 (7)
C11	0.056 (8)	0.049 (7)	0.087 (10)	−0.001 (6)	−0.028 (7)	−0.024 (7)
C12	0.081 (10)	0.058 (8)	0.102 (12)	−0.011 (7)	−0.041 (9)	−0.029 (8)
C13	0.072 (9)	0.075 (9)	0.080 (10)	0.000 (8)	−0.034 (8)	−0.020 (8)
C14	0.079 (11)	0.084 (10)	0.085 (11)	0.004 (8)	−0.036 (9)	−0.016 (9)
C15	0.086 (11)	0.088 (11)	0.088 (12)	0.000 (9)	−0.032 (10)	−0.015 (9)
C16	0.095 (13)	0.098 (12)	0.094 (13)	−0.002 (10)	−0.030 (11)	−0.014 (10)
C17	0.102 (14)	0.103 (13)	0.099 (14)	−0.003 (11)	−0.028 (11)	−0.012 (11)
C18	0.109 (15)	0.112 (14)	0.103 (15)	−0.006 (12)	−0.026 (12)	−0.011 (12)
C19	0.118 (17)	0.122 (16)	0.109 (16)	−0.007 (13)	−0.022 (13)	−0.008 (13)
C20	0.14 (2)	0.15 (2)	0.121 (18)	−0.015 (16)	−0.015 (15)	−0.012 (15)
C21	0.085 (12)	0.093 (12)	0.103 (13)	−0.013 (10)	−0.011 (10)	−0.015 (10)
C22	0.109 (15)	0.117 (16)	0.120 (17)	−0.015 (13)	−0.002 (13)	−0.005 (13)
C23	0.112 (16)	0.122 (16)	0.120 (18)	−0.017 (13)	−0.005 (14)	−0.005 (14)
C24	0.109 (16)	0.124 (16)	0.119 (18)	−0.017 (13)	−0.008 (14)	−0.007 (14)
C25	0.116 (17)	0.130 (17)	0.122 (18)	−0.016 (14)	−0.008 (14)	−0.003 (15)
C26	0.116 (17)	0.130 (17)	0.120 (18)	−0.015 (14)	−0.007 (14)	−0.006 (15)
C27	0.131 (19)	0.14 (2)	0.13 (2)	−0.016 (16)	−0.008 (16)	−0.004 (17)
C28	0.15 (2)	0.17 (2)	0.14 (2)	−0.020 (18)	−0.001 (18)	−0.008 (19)
C29	0.066 (9)	0.067 (8)	0.080 (10)	−0.002 (7)	−0.029 (8)	−0.019 (7)
C30	0.080 (11)	0.081 (10)	0.089 (12)	0.000 (8)	−0.029 (9)	−0.015 (9)
C31	0.091 (12)	0.089 (11)	0.090 (12)	−0.006 (9)	−0.031 (10)	−0.014 (10)
C32	0.101 (14)	0.098 (13)	0.098 (14)	−0.003 (11)	−0.026 (11)	−0.010 (11)
C33	0.108 (15)	0.108 (14)	0.103 (15)	−0.007 (12)	−0.026 (12)	−0.008 (12)
C34	0.120 (16)	0.118 (16)	0.111 (16)	−0.006 (13)	−0.023 (13)	−0.007 (13)
C35	0.126 (18)	0.128 (17)	0.117 (18)	−0.006 (14)	−0.017 (14)	−0.004 (14)
C36	0.16 (2)	0.17 (2)	0.14 (2)	−0.017 (19)	−0.009 (18)	−0.005 (18)
C37	0.056 (8)	0.062 (8)	0.075 (9)	−0.005 (6)	−0.026 (7)	−0.022 (7)
C38	0.066 (9)	0.075 (9)	0.081 (11)	−0.005 (8)	−0.021 (8)	−0.018 (8)
C39	0.079 (11)	0.094 (11)	0.083 (12)	−0.011 (9)	−0.019 (9)	−0.017 (9)
C40	0.093 (13)	0.108 (14)	0.097 (14)	−0.011 (11)	−0.014 (11)	−0.009 (11)
C41	0.100 (14)	0.115 (14)	0.098 (15)	−0.013 (11)	−0.012 (12)	−0.011 (12)
C42	0.108 (15)	0.125 (16)	0.106 (16)	−0.013 (13)	−0.010 (13)	−0.006 (13)
C43	0.115 (17)	0.135 (18)	0.108 (17)	−0.015 (14)	−0.009 (14)	−0.007 (14)
C44	0.14 (2)	0.18 (2)	0.13 (2)	−0.017 (18)	0.002 (17)	0.002 (17)

### Geometric parameters (Å, °)

**Table:**

Sn1—O5	1.993 (6)	C21—H21B	0.9700
Sn1—C21	2.133 (16)	C22—C23	1.50 (2)
Sn1—C13	2.135 (13)	C22—H22A	0.9700
Sn1—O4^i^	2.158 (7)	C22—H22B	0.9700
Sn1—O1	2.202 (6)	C23—C24	1.47 (2)
Sn2—C37	2.090 (12)	C23—H23A	0.9700
Sn2—C29	2.093 (13)	C23—H23B	0.9700
Sn2—O5^i^	2.111 (6)	C24—C25	1.52 (2)
Sn2—O5	2.151 (6)	C24—H24A	0.9700
Sn2—O1	2.455 (7)	C24—H24B	0.9700
Sn2—O3	2.472 (7)	C25—C26	1.49 (3)
Sn2—N1	2.829 (9)	C25—H25A	0.9700
N1—C2	1.300 (13)	C25—H25B	0.9700
N1—C5	1.334 (13)	C26—C27	1.51 (3)
N2—C4	1.313 (14)	C26—H26A	0.9700
N2—C3	1.343 (14)	C26—H26B	0.9700
N3—C8	1.334 (13)	C27—C28	1.49 (3)
N3—C11	1.345 (14)	C27—H27A	0.9700
N4—C10	1.229 (15)	C27—H27B	0.9700
N4—C9	1.326 (15)	C28—H28A	0.9600
O1—C1	1.363 (13)	C28—H28B	0.9600
O2—C1	1.189 (12)	C28—H28C	0.9600
O3—C7	1.231 (12)	C29—C30	1.503 (19)
O4—C7	1.326 (12)	C29—H29A	0.9700
O4—Sn1^i^	2.158 (7)	C29—H29B	0.9700
O5—Sn2^i^	2.111 (6)	C30—C31	1.532 (19)
C1—C2	1.500 (14)	C30—H30A	0.9700
C2—C3	1.418 (16)	C30—H30B	0.9700
C3—H3	0.9300	C31—C32	1.50 (2)
C4—C5	1.336 (16)	C31—H31A	0.9700
C4—C6	1.504 (15)	C31—H31B	0.9700
C5—H5	0.9300	C32—C33	1.52 (2)
C6—H6A	0.9600	C32—H32A	0.9700
C6—H6B	0.9600	C32—H32B	0.9700
C6—H6C	0.9600	C33—C34	1.49 (2)
C7—C8	1.411 (14)	C33—H33A	0.9700
C8—C9	1.370 (15)	C33—H33B	0.9700
C9—H9	0.9300	C34—C35	1.55 (2)
C10—C11	1.395 (16)	C34—H34A	0.9700
C10—C12	1.478 (15)	C34—H34B	0.9700
C11—H11	0.9300	C35—C36	1.52 (3)
C12—H12A	0.9600	C35—H35A	0.9700
C12—H12B	0.9600	C35—H35B	0.9700
C12—H12C	0.9600	C36—H36A	0.9600
C13—C14	1.508 (19)	C36—H36B	0.9600
C13—H13A	0.9700	C36—H36C	0.9600
C13—H13B	0.9700	C37—C38	1.500 (17)
C14—C15	1.526 (19)	C37—H37A	0.9700
C14—H14A	0.9700	C37—H37B	0.9700
C14—H14B	0.9700	C38—C39	1.503 (19)
C15—C16	1.52 (2)	C38—H38A	0.9700
C15—H15A	0.9700	C38—H38B	0.9700
C15—H15B	0.9700	C39—C40	1.48 (2)
C16—C17	1.51 (2)	C39—H39A	0.9700
C16—H16A	0.9700	C39—H39B	0.9700
C16—H16B	0.9700	C40—C41	1.50 (2)
C17—C18	1.52 (2)	C40—H40A	0.9700
C17—H17A	0.9700	C40—H40B	0.9700
C17—H17B	0.9700	C41—C42	1.48 (2)
C18—C19	1.53 (2)	C41—H41A	0.9700
C18—H18A	0.9700	C41—H41B	0.9700
C18—H18B	0.9700	C42—C43	1.52 (2)
C19—C20	1.53 (2)	C42—H42A	0.9700
C19—H19A	0.9700	C42—H42B	0.9700
C19—H19B	0.9700	C43—C44	1.46 (2)
C20—H20A	0.9600	C43—H43A	0.9700
C20—H20B	0.9600	C43—H43B	0.9700
C20—H20C	0.9600	C44—H44A	0.9600
C21—C22	1.48 (2)	C44—H44B	0.9600
C21—H21A	0.9700	C44—H44C	0.9600
			
O5—Sn1—C21	114.0 (5)	H21A—C21—H21B	107.1
O5—Sn1—C13	116.2 (4)	C21—C22—C23	115.4 (18)
C21—Sn1—C13	129.3 (6)	C21—C22—H22A	108.4
O5—Sn1—O4^i^	92.8 (3)	C23—C22—H22A	108.4
C21—Sn1—O4^i^	95.7 (5)	C21—C22—H22B	108.4
C13—Sn1—O4^i^	88.1 (4)	C23—C22—H22B	108.4
O5—Sn1—O1	75.7 (2)	H22A—C22—H22B	107.5
C21—Sn1—O1	92.0 (5)	C24—C23—C22	117.1 (19)
C13—Sn1—O1	94.2 (4)	C24—C23—H23A	108.0
O4^i^—Sn1—O1	168.0 (3)	C22—C23—H23A	108.0
C37—Sn2—C29	152.6 (5)	C24—C23—H23B	108.0
C37—Sn2—O5^i^	96.3 (4)	C22—C23—H23B	108.0
C29—Sn2—O5^i^	104.8 (4)	H23A—C23—H23B	107.3
C37—Sn2—O5	102.9 (4)	C23—C24—C25	113.4 (19)
C29—Sn2—O5	99.4 (4)	C23—C24—H24A	108.9
O5^i^—Sn2—O5	74.7 (3)	C25—C24—H24A	108.9
C37—Sn2—O1	91.0 (4)	C23—C24—H24B	108.9
C29—Sn2—O1	82.9 (4)	C25—C24—H24B	108.9
O5^i^—Sn2—O1	142.4 (2)	H24A—C24—H24B	107.7
O5—Sn2—O1	67.8 (2)	C26—C25—C24	114.3 (19)
C37—Sn2—O3	83.7 (4)	C26—C25—H25A	108.7
C29—Sn2—O3	81.0 (4)	C24—C25—H25A	108.7
O5^i^—Sn2—O3	84.7 (2)	C26—C25—H25B	108.7
O5—Sn2—O3	158.9 (3)	C24—C25—H25B	108.7
O1—Sn2—O3	132.8 (2)	H25A—C25—H25B	107.6
C37—Sn2—N1	78.1 (4)	C25—C26—C27	113 (2)
C29—Sn2—N1	75.8 (4)	C25—C26—H26A	108.9
O5^i^—Sn2—N1	158.1 (2)	C27—C26—H26A	108.9
O5—Sn2—N1	127.1 (2)	C25—C26—H26B	108.9
O1—Sn2—N1	59.3 (2)	C27—C26—H26B	108.9
O3—Sn2—N1	73.7 (2)	H26A—C26—H26B	107.7
C2—N1—C5	114.8 (10)	C28—C27—C26	115 (2)
C2—N1—Sn2	115.7 (7)	C28—C27—H27A	108.5
C5—N1—Sn2	129.5 (7)	C26—C27—H27A	108.5
C4—N2—C3	116.1 (10)	C28—C27—H27B	108.5
C8—N3—C11	116.2 (9)	C26—C27—H27B	108.5
C10—N4—C9	117.3 (11)	H27A—C27—H27B	107.5
C1—O1—Sn1	125.7 (6)	C27—C28—H28A	109.5
C1—O1—Sn2	132.8 (6)	C27—C28—H28B	109.5
Sn1—O1—Sn2	98.3 (3)	H28A—C28—H28B	109.5
C7—O3—Sn2	135.6 (7)	C27—C28—H28C	109.5
C7—O4—Sn1^i^	137.4 (7)	H28A—C28—H28C	109.5
Sn1—O5—Sn2^i^	137.1 (3)	H28B—C28—H28C	109.5
Sn1—O5—Sn2	116.6 (3)	C30—C29—Sn2	120.7 (9)
Sn2^i^—O5—Sn2	105.3 (3)	C30—C29—H29A	107.2
O2—C1—O1	122.6 (9)	Sn2—C29—H29A	107.2
O2—C1—C2	127.3 (11)	C30—C29—H29B	107.2
O1—C1—C2	109.4 (9)	Sn2—C29—H29B	107.2
N1—C2—C3	121.7 (10)	H29A—C29—H29B	106.8
N1—C2—C1	120.5 (11)	C29—C30—C31	112.8 (13)
C3—C2—C1	117.5 (10)	C29—C30—H30A	109.0
N2—C3—C2	120.7 (11)	C31—C30—H30A	109.0
N2—C3—H3	119.6	C29—C30—H30B	109.0
C2—C3—H3	119.6	C31—C30—H30B	109.0
N2—C4—C5	121.5 (10)	H30A—C30—H30B	107.8
N2—C4—C6	116.8 (11)	C32—C31—C30	114.3 (14)
C5—C4—C6	121.6 (11)	C32—C31—H31A	108.7
N1—C5—C4	125.0 (11)	C30—C31—H31A	108.7
N1—C5—H5	117.5	C32—C31—H31B	108.7
C4—C5—H5	117.5	C30—C31—H31B	108.7
C4—C6—H6A	109.5	H31A—C31—H31B	107.6
C4—C6—H6B	109.5	C31—C32—C33	112.7 (15)
H6A—C6—H6B	109.5	C31—C32—H32A	109.0
C4—C6—H6C	109.5	C33—C32—H32A	109.0
H6A—C6—H6C	109.5	C31—C32—H32B	109.0
H6B—C6—H6C	109.5	C33—C32—H32B	109.0
O3—C7—O4	118.4 (9)	H32A—C32—H32B	107.8
O3—C7—C8	125.3 (10)	C34—C33—C32	114.6 (17)
O4—C7—C8	116.1 (9)	C34—C33—H33A	108.6
N3—C8—C9	116.8 (9)	C32—C33—H33A	108.6
N3—C8—C7	117.2 (10)	C34—C33—H33B	108.6
C9—C8—C7	126.0 (11)	C32—C33—H33B	108.6
N4—C9—C8	126.0 (11)	H33A—C33—H33B	107.6
N4—C9—H9	117.0	C33—C34—C35	110.9 (17)
C8—C9—H9	117.0	C33—C34—H34A	109.5
N4—C10—C11	120.5 (11)	C35—C34—H34A	109.5
N4—C10—C12	117.9 (12)	C33—C34—H34B	109.5
C11—C10—C12	121.6 (12)	C35—C34—H34B	109.5
N3—C11—C10	123.1 (11)	H34A—C34—H34B	108.0
N3—C11—H11	118.4	C36—C35—C34	109.5 (19)
C10—C11—H11	118.4	C36—C35—H35A	109.8
C10—C12—H12A	109.5	C34—C35—H35A	109.8
C10—C12—H12B	109.5	C36—C35—H35B	109.8
H12A—C12—H12B	109.5	C34—C35—H35B	109.8
C10—C12—H12C	109.5	H35A—C35—H35B	108.2
H12A—C12—H12C	109.5	C35—C36—H36A	109.5
H12B—C12—H12C	109.5	C35—C36—H36B	109.5
C14—C13—Sn1	114.5 (9)	H36A—C36—H36B	109.5
C14—C13—H13A	108.6	C35—C36—H36C	109.5
Sn1—C13—H13A	108.6	H36A—C36—H36C	109.5
C14—C13—H13B	108.6	H36B—C36—H36C	109.5
Sn1—C13—H13B	108.6	C38—C37—Sn2	123.2 (9)
H13A—C13—H13B	107.6	C38—C37—H37A	106.5
C13—C14—C15	114.1 (13)	Sn2—C37—H37A	106.5
C13—C14—H14A	108.7	C38—C37—H37B	106.5
C15—C14—H14A	108.7	Sn2—C37—H37B	106.5
C13—C14—H14B	108.7	H37A—C37—H37B	106.5
C15—C14—H14B	108.7	C37—C38—C39	113.7 (12)
H14A—C14—H14B	107.6	C37—C38—H38A	108.8
C16—C15—C14	112.7 (13)	C39—C38—H38A	108.8
C16—C15—H15A	109.0	C37—C38—H38B	108.8
C14—C15—H15A	109.0	C39—C38—H38B	108.8
C16—C15—H15B	109.0	H38A—C38—H38B	107.7
C14—C15—H15B	109.0	C40—C39—C38	118.6 (14)
H15A—C15—H15B	107.8	C40—C39—H39A	107.7
C17—C16—C15	113.2 (14)	C38—C39—H39A	107.7
C17—C16—H16A	108.9	C40—C39—H39B	107.7
C15—C16—H16A	108.9	C38—C39—H39B	107.7
C17—C16—H16B	108.9	H39A—C39—H39B	107.1
C15—C16—H16B	108.9	C39—C40—C41	115.1 (15)
H16A—C16—H16B	107.7	C39—C40—H40A	108.5
C16—C17—C18	112.6 (16)	C41—C40—H40A	108.5
C16—C17—H17A	109.1	C39—C40—H40B	108.5
C18—C17—H17A	109.1	C41—C40—H40B	108.5
C16—C17—H17B	109.1	H40A—C40—H40B	107.5
C18—C17—H17B	109.1	C42—C41—C40	118.0 (17)
H17A—C17—H17B	107.8	C42—C41—H41A	107.8
C17—C18—C19	111.0 (16)	C40—C41—H41A	107.8
C17—C18—H18A	109.4	C42—C41—H41B	107.8
C19—C18—H18A	109.4	C40—C41—H41B	107.8
C17—C18—H18B	109.4	H41A—C41—H41B	107.2
C19—C18—H18B	109.4	C41—C42—C43	114.4 (17)
H18A—C18—H18B	108.0	C41—C42—H42A	108.7
C20—C19—C18	109.9 (17)	C43—C42—H42A	108.7
C20—C19—H19A	109.7	C41—C42—H42B	108.7
C18—C19—H19A	109.7	C43—C42—H42B	108.7
C20—C19—H19B	109.7	H42A—C42—H42B	107.6
C18—C19—H19B	109.7	C44—C43—C42	116 (2)
H19A—C19—H19B	108.2	C44—C43—H43A	108.3
C19—C20—H20A	109.5	C42—C43—H43A	108.3
C19—C20—H20B	109.5	C44—C43—H43B	108.3
H20A—C20—H20B	109.5	C42—C43—H43B	108.3
C19—C20—H20C	109.5	H43A—C43—H43B	107.4
H20A—C20—H20C	109.5	C43—C44—H44A	109.5
H20B—C20—H20C	109.5	C43—C44—H44B	109.5
C22—C21—Sn1	118.2 (13)	H44A—C44—H44B	109.5
C22—C21—H21A	107.8	C43—C44—H44C	109.5
Sn1—C21—H21A	107.8	H44A—C44—H44C	109.5
C22—C21—H21B	107.8	H44B—C44—H44C	109.5
Sn1—C21—H21B	107.8		

Symmetry codes: (i) −*x*+1, −*y*+2, −*z*+2.
